# Human-like intuitive behavior and reasoning biases emerged in large language models but disappeared in ChatGPT

**DOI:** 10.1038/s43588-023-00527-x

**Published:** 2023-10-05

**Authors:** Thilo Hagendorff, Sarah Fabi, Michal Kosinski

**Affiliations:** 1 University of Stuttgart, Stuttgart, Germany; 2 University of California San Diego, San Diego, CA USA; 3 Stanford University, Stanford, CA USA

**Keywords:** Psychology, Computer science, Computational science

## Abstract

We design a battery of semantic illusions and cognitive reflection tests, aimed to elicit intuitive yet erroneous responses. We administer these tasks, traditionally used to study reasoning and decision-making in humans, to OpenAI’s generative pre-trained transformer model family. The results show that as the models expand in size and linguistic proficiency they increasingly display human-like intuitive system 1 thinking and associated cognitive errors. This pattern shifts notably with the introduction of ChatGPT models, which tend to respond correctly, avoiding the traps embedded in the tasks. Both ChatGPT-3.5 and 4 utilize the input–output context window to engage in chain-of-thought reasoning, reminiscent of how people use notepads to support their system 2 thinking. Yet, they remain accurate even when prevented from engaging in chain-of-thought reasoning, indicating that their system-1-like next-word generation processes are more accurate than those of older models. Our findings highlight the value of applying psychological methodologies to study large language models, as this can uncover previously undetected emergent characteristics.

## Abstract (web summary)

The reasoning capabilities of OpenAI’s generative pre-trained transformer family were tested using semantic illusions and cognitive reflection tests that are typically used in human studies. While early models were prone to human-like cognitive errors, ChatGPT decisively outperformed humans, avoiding the cognitive traps embedded in the tasks.

## Main

As the range of applications for large language models (LLMs) rapidly expands, it is of paramount importance to understand the mechanisms through which LLMs reason and make decisions. Recent research has revealed that with the increasing complexity of LLMs they exhibit a multitude of skills and properties, some of which were not anticipated or intended by their creators^1,2^. Among these newfound abilities are the capacity to generate computer code, tackle mathematical problems, learn from examples, engage in introspection, carry out multistep reasoning, solve theory of mind tasks, deceive other agents and a plethora of other skills^3–6^. In this work, we aim to explore reasoning capabilities in the family of generative pre-trained transformer (GPT) models by OpenAI, while shedding light on the intricacies of their cognitive processes.

Research on humans often distinguishes between two broad categories of reasoning or—more broadly—cognitive processes: systems 1 and 2^7,8^. System 1 processes are fast, automatic and instinctual. They often involve heuristics, or mental shortcuts, which enable quick judgments and decisions without conscious effort. System 1 is essential for everyday functioning, as it allows humans to navigate their environments and make rapid decisions with minimal effort. System 2 processes, on the other hand, are deliberate and require conscious effort. This system is employed in logical reasoning, critical thinking and problem-solving. System 2 processes are slower and more resource intensive, but they are also more accurate and less susceptible to bias.

On the surface, current-day LLMs seem to be system 1 thinkers: the input text is processed by consecutive layers of neurons to produce a distribution of probabilities of all possible single-token (word) completions. This process is automatic and unidirectional, and involves a single wave of propagation through the neural network for each consecutive predicted word. Yet, past research and the results presented here suggest that, like humans, LLMs can also engage in system-2-like cognitive processes^4^. While generating each consecutive word, LLMs re-read their context window, including the task provided by a user, as well as the words they have thus far generated. As a result, LLMs can employ their context window as a form of an external short-term memory to engage in chain-of-thought reasoning, re-examine the starting assumptions, estimate partial solutions or test alternative approaches. This is akin to how people use notepads to solve mathematical problems or write essays to sharpen and develop their arguments.

In this work, we build on psychological research on human reasoning and decision-making to explore system 1 and 2 processes in LLMs. We examine the performance of humans (*n* = 455) and ten OpenAI LLMs (ranging from GPT-1 to ChatGPT-4^3,9–11^) using tasks typically employed to test reasoning and decision-making in humans: cognitive reflection test (CRT)^12^ tasks and semantic illusions^13^ (see Supplementary Information and Supplementary Table 1 for more details). The CRT comprises three types of mathematical tasks that appear to be simpler than they really are, thus triggering an intuitive but incorrect system 1 response. CRT type 1 tasks, such as the widely known ‘A bat and a ball’ task, use a ‘more than’ phrase to trick participants into subtracting two of the values rather than solving a somewhat more complex equation. Type 2 tasks exploit people’s tendency to complete a numerical triplet series, such as five machines making five widgets in five minutes because two machines make two widgets in two minutes. Type 3 tasks describe an exponential process but trick the participants into treating it as linear. Solving CRT tasks correctly requires engaging in deliberate system 2 reasoning or possessing well developed system 1 intuitions. Semantic illusions are questions containing a disguised error aimed at triggering an intuitive but incorrect system 1 response. In the well known Moses Illusion^13^, for example, participants tend to be tricked into claiming that Moses took two animals of each kind on the Ark (when in fact it was Noah).

We address some of the limitations of past studies. First, while past research focused on a single model (GPT-3), we study reasoning capabilities across a range of models of different sizes and complexities. Second, as solving the CRT tasks requires mathematical abilities, LLMs’ performance could be limited by their mathematical skills. To address this issue, we complement the CRT tasks with semantic illusions that do not rely on mathematical skills. Third, past research relied on three CRT tasks copied verbatim from human studies^14^. This is problematic, as observing LLMs’ performance on three CRT tasks does not allow for meaningful statistical comparisons. Moreover, these tasks (as well as their solutions) were likely present in the LLMs’ training data. To circumvent these issues, we designed 50 bespoke versions of each type of task (200 in total).

For brevity and convenience, we use words such as ‘behavior’, ‘intuition’, ‘deliberation’ or ‘ability’ when referring to LLMs, yet we do not mean to equate artificial intelligence (AI) and human cognitive processes. While AI’s outputs are often similar to ones produced by humans, it typically operates in fundamentally different ways.

**Fig. 1:**
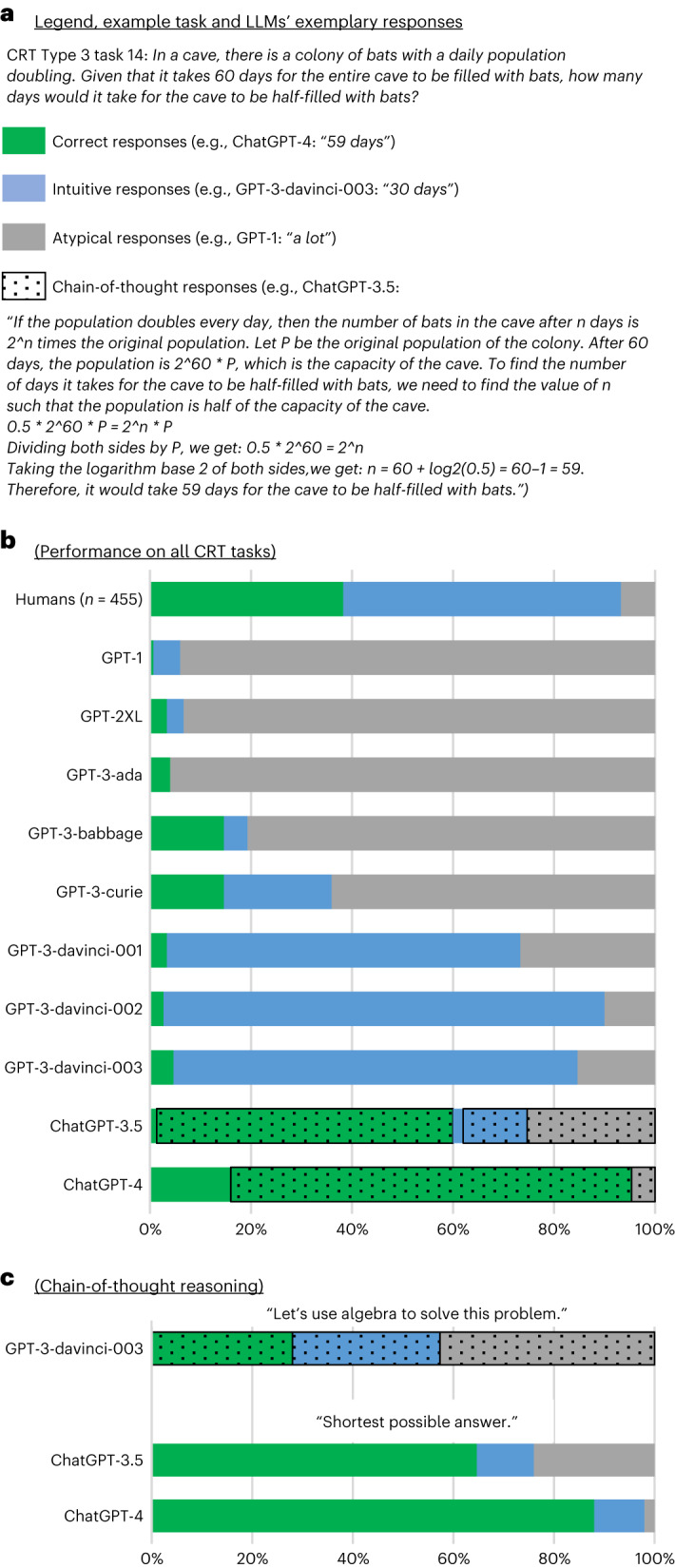
Human and LLM performance on the CRT tasks. **a**, Exemplary responses to one of the CRT tasks, categorized as correct, intuitive (but incorrect) and atypical (that is, all other incorrect responses). Within each category, the responses that were preceded by written chain-of-thought reasoning were additionally labeled as ‘chain-of-thought responses’. **b**, Human and LLM performance on 150 CRT tasks. **c**, LLMs’ responses when instructed to engage or prevented from engaging in chain-of-thought reasoning. The data source file includes 95% confidence intervals. Source data

First, we present the results of study 1: the cognitive reflection test. To help the reader interpret the results, we discuss them in the context of LLMs’ exemplary responses to one of the CRT tasks (Fig. 1a). The correct response to this task is ‘59 days’, but it was designed to appear easier than it really is, tempting participants to simply divide the total time by two, triggering an intuitive (but incorrect) response of ‘30 days’.

The performance of humans and LLMs across 150 CRT tasks is presented in Fig. 1b. There are four distinct trends. First, most of the responses of early and smaller LLMs (up until GPT-3-curie) were atypical. This category includes responses that were evasive (for example, GPT-1’s response “a lot”), which indicated failure to comprehend the task (for example, GPT-2XL’s response “The colony would take 60 days to double in size”) or which were incorrect in ways different from one that the task was designed to trigger (for example, GPT-3-babbage’s response: “It would take about 10 days for the cave to be half-filled with bats”). Moreover, while 15% of responses of both GPT-3-babbage and GPT-3-curie were categorized as correct, they seemed accidental: all but one were given CRT type 2 tasks, which can be solved by simply repeating the number mentioned most frequently in the prompt—which these models tended to naively do in this and other tasks.

Second, as the models grew larger and their ability to comprehend the task increased, atypical responses were replaced by intuitive (but incorrect) responses, which the tasks were designed to trigger (for example, GPT-3-davinci-003: “30 days”). These constituted below 5% of responses of early models (up to GPT-3-babbage) and increased to 21% for GPT-3-curie (difference ($\delta$) = 16%; *χ*^2^(1) = 16.98; *P* < 0.001) and to 70%–90% for the GPT-3-davinci family (δ ≥ 49%; *χ*^2^(1) ≥ 69.64; *P* < 0.001), a fraction much higher than observed in humans (55%; δ ≥ 15%; *χ*^2^(1) ≥ 11.79; *P* < 0.001).

In humans, intuitive but incorrect responses are interpreted as evidence of system 1 reasoning and failure to engage system 2, but they could also stem from deliberate—yet erroneous—system 2 reasoning. The generative process behind the LLMs’ responses is less ambiguous. As we discuss in the introduction, current-day LLMs lack the built-in cognitive infrastructure necessary to internally engage in system 2 processes. Consequently, their intuitive responses can only stem from a system-1-like process.

Importantly, LLMs’ intuitive responses are unlikely to be driven by insufficient mathematical ability. First, previous research has shown that LLMs can solve basic mathematical problems^1^. Second, intuitive responses to the CRT type 1 and 3 tasks also require solving a simple equation (for example, how much is ‘half of 60’; responding intuitively to the CRT type 2 tasks requires no computation). Moreover, as we show in study 3, GPT-3-davinci-003’s performance can be substantially improved by presenting it with training examples.

Third, LLMs’ strong tendency to respond intuitively stops abruptly with the arrival of ChatGPT. The fraction of correct responses was equal to 59% for ChatGPT-3.5 and 96% for ChatGPT-4. This is much higher than the 5% of tasks solved correctly by GPT-3-davinci-003, an otherwise very apt model (δ ≥ 54%; *χ*^2^(1) ≥ 102.44; *P* < 0.001), or 38% achieved by humans (δ ≥ 21%; *χ*^2^(1) ≥ 25.60; *P* < 0.001). ChatGPT’s tendency to respond correctly was accompanied by a substantial drop in its tendency to respond intuitively: 15% for ChatGPT-3.5 and 0% for ChatGPT-4 versus 80% for GPT-3-davinci-003 (δ ≥ 65%; *χ*^2^(1) ≥ 125.81; *P* < 0.001) and 55% for humans (δ ≥ 40%; *χ*^2^(1) ≥ 86.30; *P* < 0.001).

A closer look at ChatGPT models’ responses reveals that the marked increase in their performance was accompanied by a novel response style. While pre-ChatGPT LLMs responded with brief phrases or single sentences (for example, GPT-3-davinci-003: “30 days”), 97% of ChatGPT-3.5’s responses and 85% of ChatGPT-4.5’s responses included some form of chain-of-thought reasoning (Fig. 1a).

As we discussed before, there is nothing deliberate in how ChatGPT-3.5 and other current-day LLMs generate the next word. Yet, each time the word is generated, an LLM re-reads the task and the response it has generated so far, combining system-1-like next-word generation into something resembling a system 2 process: generating a strategy needed to solve the task, dividing the task into more tractable subtasks and solving them one by one. This is akin to how humans use a notebook to solve mathematical tasks without the need to process them in their short-term memory.

Next, we show that chain-of-thought responses (study 2) not only resemble but also serve as system 2 processes, in line with previous studies showing that instructing LLMs to think step by step improves their ability to solve various tasks^4^.

We first show that GPT-3-davinci-003’s accuracy increases when it is instructed to engage in chain-of-thought reasoning. We present it with the CRT tasks suffixed with “Let’s use algebra to solve this problem”. The results presented in Fig. 1c show that our manipulation was successful: the fraction of chain-of-thought responses increased from 0% in study 1 to 100% (δ = 100%; *χ*^2^(1) = 147.01; *P* < 0.001). The model seemed to design and execute a task-solving strategy. Most of the time, this strategy was poorly conceived or executed, leading to the increase of atypical responses from 15% to 43% (δ = 28%; *χ*^2^(1) = 14.72; *P* < 0.001). Yet, in other cases, the strategy was sound, boosting the fraction of correct responses from 5% to 28% (δ = 23%; *χ*^2^(1) = 28.20; *P* < 0.001) and reducing the model’s tendency to fall for the trap embedded in the task: intuitive responses dropped from 80% to 29% (δ = 51%; *χ*^2^(1) = 75.66; *P* < 0.001).

Next, we show that preventing the model from engaging in chain-of-thought reasoning can decrease its ability to solve the tasks. We presented ChatGPT models with the CRT tasks suffixed with “Provide the shortest possible answer (for example, ‘$2’ or ‘1 week’), do not explain your reasoning”. The results presented in Fig. 1 show that our manipulation was again successful: the fraction of chain-of-thought responses fell from 97% to 0% for ChatGPT-3.5 (δ = 97%; *χ*^2^(1) = 276.79; *P* < 0.001) and from 84% to 0% for ChatGPT-4 (δ = 84%; *χ*^2^(1) = 213.81; *P* < 0.001). The fraction of correct responses did not change for ChatGPT-3.5 (δ = 4%; *χ*^2^(1) = 0.47; *P* = 0.49). For ChatGPT-4, it fell from 95% to 88% (δ = 7%; *χ*^2^(1) = 4.36; *P* < 0.05), accompanied by an increase in intuitive responses from 0% to 10% (δ = 10%; *χ*^2^(1) = 13.75; *P* < 0.001).

The results of study 2 suggest that chain-of-thought reasoning helps LLMs to avoid falling for the traps embedded in the CRT tasks and improves their ability to solve them correctly. Yet, they also reveal that ChatGPT models could solve the great majority of the CRT tasks even when forced to provide a system-1-like response. This is consistent with ChatGPT-4’s performance in study 1, where it solved 24% of the CRT task without using chain-of-thought reasoning.

In humans, this would be taken as evidence of a well developed intuition stemming from previous exposure to similar tasks^15^ (although the persistence and size of this effect is disputed^16^). Here we show results suggesting that the same applies to LLMs. This is in line with past results showing that LLMs can learn, even from a single example^3^.

As ChatGPT models already seem to possess well developed intuition, we attempt to improve the system-1-like responses of GPT-3-davinci-003 (study 3). We present it with each of the CRT tasks, each time preceding this with 0 to 49 remaining tasks of the same type, accompanied by the correct solution. The CRT tasks of the same type are semantically very similar, enabling the model to develop system-1 intuitions akin to that expressed by the ChatGPT model family.

**Extended Data Fig. 1:**
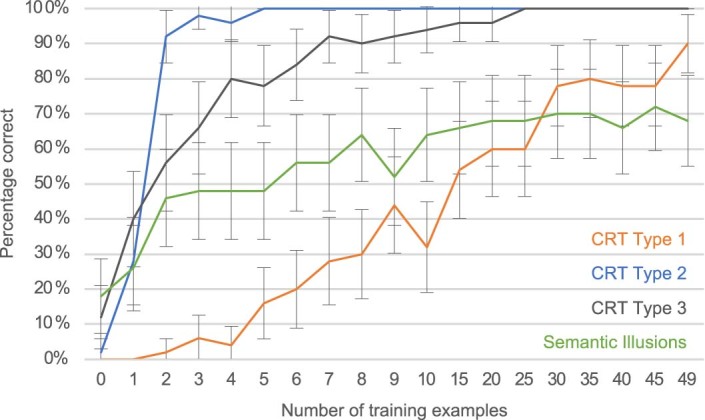
Learning curves. Change in the fraction of GPT-3-davinci-003’s correct responses against the number of training examples that the task was prefixed with. Error bars represent 95% confidence intervals. Source data

The results presented in Extended Data Fig. 1 show that GPT-3-davinci-003’s ability to answer correctly (rather than intuitively) increased with each additional example. The fastest gains were observed for the CRT type 2 tasks, where the accuracy increased from 2% to 92% after two examples (δ = 90%; *χ*^2^(1) = 77.72; *P* < 0.001). This is to be expected, as they can be solved correctly by simply repeating the duration listed in the task. The CRT type 3 tasks, solvable by reporting the total time minus one unit, proved to be somewhat more complex: the accuracy increased from 12% to 92% after seven training examples (δ = 80%; *χ*^2^(1) = 60.94; *P* < 0.001). It took most examples to develop the model’s intuition to solve the CRT type 1 tasks, where the correct answer is equal to $\frac{{\mathrm{total}}\; {\mathrm{price}}-{\mathrm{known}}\; {\mathrm{price}}}{2}$. However, even here, the model’s accuracy increased from 0% to 78% after 30 examples (δ = 78%; *χ*^2^(1) = 60.70; *P* < 0.001).

The CRT tasks employed in studies 1–3 rely heavily on mathematical skills and are highly semantically uniform. To ensure that the results generalize beyond the CRT tasks, we replicate studies 1–3 using much more semantically diverse semantic illusions (study 4). Similarly to the CRT tasks, semantic illusions contain a disguised error aimed at triggering an intuitive but incorrect system 1 response. Unlike the CRT tasks, semantic illusions do not require mathematical skills, instead relying on participants’ general knowledge.

**Fig. 2:**
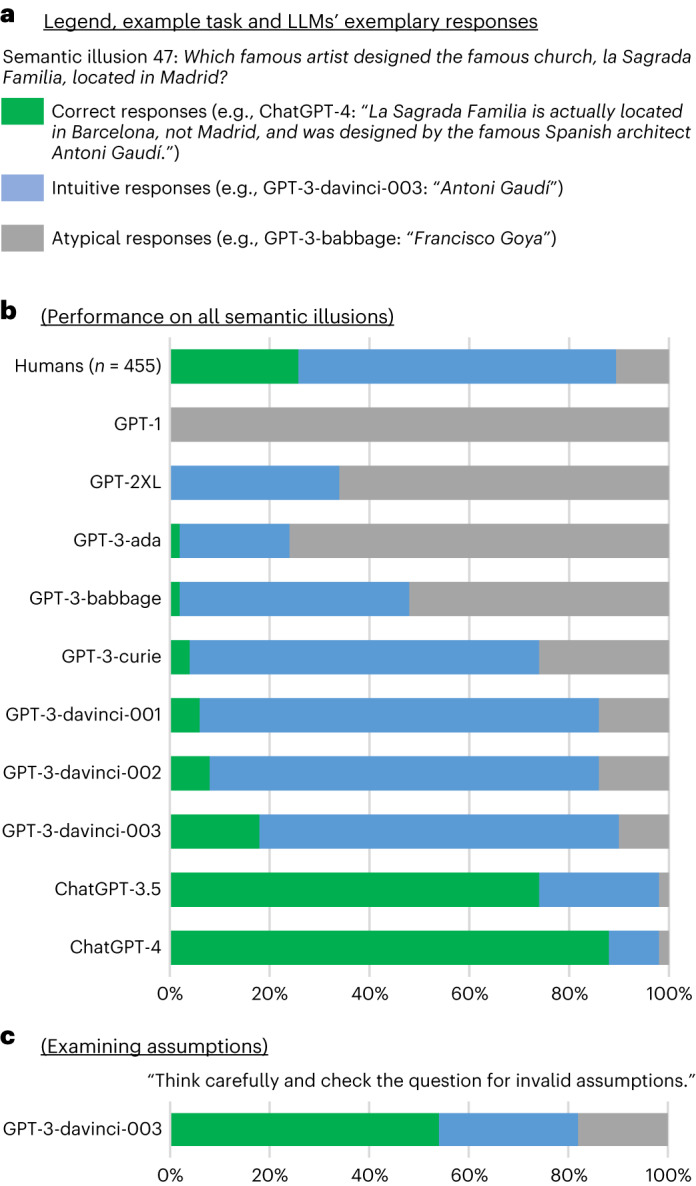
Human and LLM performance on semantic illusions. **a**. Exemplary responses to one of the semantic illusions, categorized as correct, intuitive and atypical. **b**, Human and LLM performance on 50 semantic illusions. **c**, GPT-3-davinci-003’s responses when instructed to examine the task’s assumptions. The data source file includes 95% confidence intervals. Source data

To help the reader interpret the results, we discuss them in the context of LLMs’ exemplary responses to semantic illusion 47 (Fig. 2a). As in the context of the CRT tasks, responses were divided into three categories: intuitive, correct and atypical. The question was designed to trigger an intuitive system-1 response ‘Antoni Gaudí’ while overlooking the embedded invalid assumption (la Sagrada Família is in Barcelona). Importantly, responding ‘Antoni Gaudí’ can be treated as indicative of system 1 processing only if the respondent has the knowledge necessary to recognize the error. Thus, given an intuitive response, the model was reset, and its underlying knowledge was tested using an additional question (here “Where is the famous church, la Sagrada Família, located?”; see Supplementary Information for the list of knowledge questions). Intuitive responses given by LLMs that failed this post hoc test were recategorized as atypical, along with responses revealing a further lack of necessary knowledge (for example, GPT-3-babbage: “Francisco Goya”) and nonsensical responses (for example, GPT-1: “the church of san francisco”). Responses recognizing the invalid assumption were categorized as correct.

The results presented in Fig. 2b show a pattern similar to one observed in study 1. Most of the responses of early and smaller LLMs (up to GPT-3-babbage) were atypical (gray bars), as they struggled to comprehend the question or lacked the necessary knowledge. As LLMs grew in size and overall ability, the fraction of atypical responses fell from 52% for GPT-3-babbage to 10% for GPT-3-davinci-003 (δ = 42%; *χ*^2^(1) = 18.70; *P* < 0.001). They were replaced by intuitive responses (blue bars): GPT-3-davinci-003 fell for the semantic illusion 72% of the time. As in the CRT tasks, this trend changes markedly with the introduction of ChatGPT. The fraction of correct responses increased from 18% for GPT-3-davinci-003 to 74% and 88% for ChatGPT-3.5 and ChatGPT-4, respectively (green bars; δ ≥ 56%; *χ*^2^(1) = 29.35; *P* < 0.001). As we discussed before, there is nothing deliberate in LLMs’ next-word generation process, yet this system-1-like process proved to be very apt at detecting invalid assumptions embedded in semantic illusions.

The results of studies 2 and 3 suggest that LLMs’ propensity to commit reasoning errors in the CRT tasks can be reduced by instructing them to examine the task more carefully and providing them with examples of correct solutions to similar tasks. Study 5 replicates these results in the context of semantic illusions.

We first add the suffix “Think carefully and check the question for invalid assumptions” to each semantic illusion and administer them to GPT-3-davinci-003. The results presented in Fig. 2c show that the fraction of correct responses increased threefold, from 18% in study 4 to 54% (δ = 36%; *χ*^2^(1) = 12.54; *P* < 0.001), while the fraction of intuitive responses decreased from 72% to 28% (δ = 44%; *χ*^2^(1) = 17.64; *P* < 0.001).

Next, as in study 3, we precede each semantic illusion with 0 to 49 other semantic illusions, accompanied by the correct solution. The results presented in Extended Data Fig. 1 show that GPT-3-davinci-003’s ability to answer correctly increased from 18% for zero examples to over 64% for ten and more examples (δ ≥ 46%; *χ*^2^(1) = 20.01; *P* < 0.001).

## Discussion

Our results reveal an interesting pattern. As LLMs’ ability to comprehend the tasks increases, they tend to fall for the traps embedded in the tasks. This, in humans, would be interpreted as evidence of fast, automatic and instinctual system 1 processing. The most able of the pre-ChatGPT models, GPT-3-davinci-003, decisively outpaces humans in its tendency to respond intuitively rather than correctly. Yet, this changes abruptly with the arrival of ChatGPT models. They responded correctly to a great majority of tasks, decisively outperforming humans in their ability to avoid traps embedded in the tasks.

How would we explain pre-ChatGPT models’ tendency to respond intuitively, despite their sufficient mathematical abilities and factual knowledge demonstrated in studies 3–5? As we discuss in the introduction, LLMs lack the cognitive infrastructure necessary to engage in system 2 processes, which humans may employ when answering such questions. Thus, in the absence of well developed intuition or explicit chain-of-thought reasoning, they are particularly prone to fall for the traps embedded in the tasks.

Furthermore, how would we explain the steep shift in accuracy between GPT-3 and ChatGPT? ChatGPT models tend to engage in chain-of-thought reasoning: the models use their input–output context window to develop strategies needed to solve the task, examine the starting assumptions, estimate partial solutions or test alternative approaches—in a way akin to how people use notepads to solve mathematical problems or write essays to develop their arguments. Instructing an older model (that is, GPT-3-davinci-003) to engage in chain-of-thought reasoning substantially boosts its performance.

Yet, chain-of-thought reasoning cannot be the sole explanation. ChatGPT models’ accuracy barely drops when they are prevented from engaging in chain-of-thought reasoning. This suggests that they have well developed intuitions enabling them to solve tasks without engaging system-2-like processes. This is confirmed by results showing that GPT-3-davinci-003’s performance can be substantially increased by presenting it with example tasks and their correct solutions.

Some progress is to be expected. In humans, the CRT and semantic illusions are good predictors of an ability to engage in unbiased, reflective and rational decision-making^17^, as well as overall cognitive ability^12^. Thus, LLMs’ ability to solve the CRT and semantic illusions should increase as their overall ability increases. Yet, the shift observed in this study seems to be steeper than the increase in LLMs’ overall abilities. We can only speculate on this, given that OpenAI does not provide their models in open access and only shares limited information on their technical specification and training process. First, it is unlikely that the shift was driven merely by larger model size. According to OpenAI, ChatGPT-3.5-turbo was derived from text-davinci-003 by fine-tuning it for chat. The two models are likely of similar sizes. Second, it could be that the shift was driven by the employment of reinforcement learning from human feedback^18,19^. In reinforcement learning from human feedback, human-written demonstrations on example prompts are used to train supervised learning baselines. Next, human ‘AI trainers’ rank model outputs on a larger set of prompts, and a reward model is trained to predict their preferences. This reward model is then used to fine-tune the models using Proximal Policy Optimization algorithms. While reinforcement learning from human feedback has been employed since GPT-3 text-davinci-002^19^, this procedure was enhanced in ChatGPT training: AI trainers played both sides: the user and an AI assistant^20^. Next, it is likely that ChatGPT models were exposed to sufficient CRT-like tasks in their training to be able to respond to them intuitively. Those tasks are highly semantically similar and, as illustrated by study 3, exposure to training examples can rapidly boost an LLM’s accuracy. This explanation is less likely in the context of semantic illusions, which are much more irregular and diverse. This question will hopefully be addressed by further research or more transparency in LLM development.

Next to the analysis of LLM performance on reasoning tasks, one can approach the issue from a normative perspective, asking whether phenomena of intuitive decision-making are desirable in LLMs. In the cognitive science literature, researchers stress that the notion of intuitive errors relies on a normative concept of logics and statistics, which can be inappropriate for real-world situations. Instead, decision-making processes should be evaluated in the sense of ‘ecological rationality’, meaning on the basis of how well they fit the structure of the environment in which they occur^21^. In this vein, the CRT tasks as well as semantic illusions create a ‘hostile’ test environment, which intentionally aims to mislead humans. Should LLMs perhaps go with the conversational flow and just ‘overlook’ small mistakes instead of correcting factually incorrect questions, as humans tend to do? Or should they insist on correcting mistakes (as ChatGPT models often did in our study), so as to minimize the inaccuracies, ‘hallucinated’ outputs, factual incorrectness and misinformation—a major problem in LLM use?

We list a few of the limitations of our study. First, it is limited to OpenAI’s GPT family of models. There are many other models, including non-English-language models, whose functioning should be studied. Second, our study was limited to just two types of tasks; future work should examine other tasks or real-world examples. Third, we focused on LLMs’ observable behavior; it would be useful to study the patterns of their neural activations. Finally, many of our tasks—and particularly the CRT type 1 and 2 tasks—were highly schematic. It is possible that some models encountered enough examples in their training to solve them ‘from memory’.

The progress in LLMs not only increased their capabilities, but also reduced our ability to anticipate their properties and behavior. It is increasingly difficult to study LLMs through the lenses of their architecture and hyperparameters. Instead, as we show in this work, LLMs can be studied using methods designed to investigate another capable and opaque structure, namely the human mind. Our approach falls within a quickly growing category of studies employing classic psychological tests and experiments to probe LLM ‘psychological’ processes, such as judgment, decision-making and cognitive biases^14,22–24^.

## Methods

### Tasks

Hypothesis-blind research assistants recruited on Upwork, a freelancing platform, prepared 50 semantic illusions and 50 CRT type 3 tasks. The CRT type 1 and 2 tasks were generated automatically. All tasks can be found in Supplementary Information.

### Testing LLM performance

The tasks were administered to the family of OpenAI GPT models ranging from GPT-1 to ChatGPT-4^3,9–11^. To minimize the variance in the models’ responses and thus increase the replicability of our results, the ‘temperature’ parameter was set to 0. For ChatGPT models, the default (‘You are a helpful assistant.’) system message was used. The task was prefixed by ‘Question:’ and suffixed with ‘\nAnswer:’ for other models. As specified in the main text, in some experiments, additional suffixes were added to the tasks, such as ‘Let’s use algebra to solve this problem’. The models’ response length was set to 100 tokens but was extended if needed. The responses were trimmed once they started repeating themselves or stopped responding to the task. The LLMs’ responses were reviewed and scored manually.

### Testing human performance

The same tasks were also administered to 500 human participants recruited on Prolific.io on 10 June 2023 (50% female). Each participant was presented with a random set of four tasks (one of each kind) followed by a control question inquiring whether they used a language model or another external resource; 45 participants responded positively and were excluded from the analysis. Human respondents’ performance suggests that our tasks were of similar difficulty to those used in past human studies. In the CRT, 38% of responses were correct, compared with 41% in the original study (*n* = 3,428)^9^ (δ = 3%; *χ*^2^(1) = 3.60; *P* = 0.06). In semantic illusions, 64% of participants responded intuitively, compared with 52% in the original study (*n* = 61; they did not report the fraction of correct responses (δ = 12%; *χ*^2^(1) = 2.41; *P* = 0.12))^13^.

### Statistics and reproducibility

Proportions were compared using the prop.test() function in R^25^. All statistical tests were two sided. No statistical method was used to predetermine the number of tasks. The number of human respondents was chosen to enable the detection of small effects (Cohen’s *h* = 0.2) with the power of 0.8 at the significance level of 0.05. The resulting desired number of total responses to each test (*n* = 196) was multiplied by 2.5 to account for potential dropouts.

### Ethics

The study was executed in strict adherence to ethical guidelines and standards. Our procedures were reviewed and approved by Stanford University’s institutional review board. All participants were made fully aware of the nature and objectives of the study and provided informed consent.

### Reporting summary

Further information on research design is available in the Nature Portfolio Reporting Summary linked to this article.

### Supplementary information

Supplementary Information List of models, Supplementary Table 1, list of CRT 1 tasks, list of CRT 2 tasks, list of CRT 3 tasks, list of semantic illusions, questions used to test the knowledge necessary to solve semantic illusions, and study description given to human participants.

Reporting Summary

### Source data

Source Data Fig. 1 Human and LLM performance on 150 CRT tasks.

Source Data Fig. 2 Human and LLM performance on 50 semantic illusion tasks.

Source Data Extended Data Fig. 1 Fraction of GPT-3-davinci-003’s correct responses against the number of training examples with which the task was prefixed.

## Data Availability

All datasets are publicly available at https://osf.io/w5vhp. Source data for all figures are available with this Brief Communication.

## Code availability

Our code is publicly available at https://osf.io/w5vhp (ref.^26^)^.^
