# Supplementary material for: Human-like intuitive behavior and reasoning biases emerged in large language models but disappeared in ChatGPT
Source: Nat Comput Sci. 2023 Oct 5;3(10):833–8. doi: 10.1038/s43588-023-00527-x (PMC10766525; doi:10.1038/s43588-023-00527-x)
Supplement: Supplementary file 1 — List of models, Supplementary Table 1, list of CRT 1 tasks, list of CRT 2 tasks, list of CRT 3 tasks, list of semantic illusions, questions used to test the knowledge necessary to solve semantic illusions, and study description given to human participants. [file 43588_2023_527_MOESM1_ESM.pdf]

nature computational science

Brief Communication

https://doi.org/10.1038/s43588-023-00527-x

Human-like intuitive behavior and reasoning biases emerged in large language models but disappeared in ChatGPT

In the format provided by the authors and unedited

Supplementary Information

Table of Contents

List of models (number of parameters; publication date): 2
Supplementary Table 1. The original tasks and examples of bespoke tasks written for our study. 3
List of CRT 1 tasks 4
List of CRT 2 tasks 7
List of CRT 3 tasks 10
List of Semantic Illusions 14
Questions used to test the knowledge necessary to solve semantic illusions. 16
Study description given to human participants 18

List of models (number of parameters; publication date):

ChatGPT-4 (size unknown; March 2023)

ChatGPT-3.5 (size unknown; November 2022)

GPT-3-davinci-003 (175B; November 2022)

GPT-3-davinci-002 (175B; January 2022)

GPT-3-davinci-001 (175B; May 2020)

GPT-3-curie-001 (6.7B; May 2020)

GPT-3-babbage-001 (1.3B; May 2020)

GPT-3-ada-001 (350M; May 2020)

GPT-2XL (1.5B; February 2019)

GPT-1 (117M; June 2018)

Supplementary Table 1. The original tasks and examples of bespoke tasks written for our study.

Original tasks			Our version (examples)		
	Response			Response	
Prompt	Correct	Intuitive	Prompt	Correct	Intuitive
CRT Type 1					
A bat and a ball cost $1.10 in total. The bat costs $1.00 more than the ball. How much does the ball cost?	$0.05	$0.10	A potato and a camera together cost $1.40. The potato costs $1 more than the camera. How much does the camera cost?	$0.20	$0.40
CRT Type 2					
If it takes 5 machines 5 minutes to make 5 widgets, how long would it take 100 machines to make 100 widgets?	5 minutes	100 minutes	How long does it take 4 people to tailor 4 jackets, if it takes 7 people 7 hours to tailor 7 jackets?	7 hours	4 hours
CRT Type 3					
In a lake, there is a patch of lily pads. Every day, the patch doubles in size. If it takes 48 days for the patch to cover the entire lake, how long would it take for the patch to cover half of the lake?	47 days	24 days	People are escaping from war. Each day, the total count of refugees doubles. If it takes 22 days for the entire population to evacuate, how long would it take for half of the population to do so?	21 days	11 days
Semantic Illusions					
How many animals of each kind did Moses take on the Ark?	It was Noah	Two	Where on their bodies do whales have their gills?	Whales do not have gills	On the sides of their heads

List of CRT 1 tasks

1: A pear and a fridge together cost $140. The pear costs $100 more than the fridge. How much does the fridge cost?
2: A potato and a camera together cost $1.40. The potato costs $1 more than the camera. How much does the camera cost?
3: A boat and a potato together cost $110. The boat costs $100 more than the potato. How much does the potato cost?
4: A light bulb and a pan together cost $12. The light bulb costs $10 more than the pan. How much does the pan cost?
5: A chair and a coat together cost $13. The chair costs $10 more than the coat. How much does the coat cost?
6: A tube of toothpaste and a wallet together cost $54. The tube of toothpaste costs $50 more than the wallet. How much does the wallet cost?
7: A coffee table and a mixing bowl together cost $52. The coffee table costs $50 more than the mixing bowl. How much does the mixing bowl cost?
8: A microwave oven and a flower box together cost $51. The microwave oven costs $50 more than the flower box. How much does the flower box cost?
9: A can of baby food and a helmet together cost $1.20. The can of baby food costs $1 more than the helmet. How much does the helmet cost?
10: A sculpture and a box of lipstick together cost $13. The sculpture costs $10 more than the box of lipstick. How much does the box of lipstick cost?
11: A pair of leggings and a body lotion together cost $54. The pair of leggings costs $50 more than the body lotion. How much does the body lotion cost?
12: A trampoline and a box of batteries together cost $520. The trampoline costs $500 more than the box of batteries. How much does the box of batteries cost?
13: A mouse and a can of baby food together cost $1.20. The mouse costs $1 more than the can of baby food. How much does the can of baby food cost?
14: A bottle of shampoo and a knife together cost $110. The bottle of shampoo costs $100 more than the knife. How much does the knife cost?
15: A bottle of bath salts and a set of crayons together cost $520. The bottle of bath salts costs $500 more than the set of crayons. How much does the set of crayons cost?
16: A set of wine glasses and a pencil together cost $12. The set of wine glasses costs $10 more than the pencil. How much does the pencil cost?
17: A wallet and a camera together cost $52. The wallet costs $50 more than the camera. How much does the camera cost?
18: A pair of hiking boots and a coffee table together cost $1.20. The pair of hiking boots costs $1 more than the coffee table. How much does the coffee table cost?
19: A tube of toothpaste and a pear together cost $12. The tube of toothpaste costs $10 more than the pear. How much does the pear cost?
20: A trampoline and a flower box together cost $54. The trampoline costs $50 more than the flower box. How much does the flower box cost?
21: A silicone case and a lawnmower together cost $130. The silicone case costs $100 more than the lawnmower. How much does the lawnmower cost?
22: A toy and a microwave oven together cost $5.40. The toy costs $5 more than the microwave oven. How much does the microwave oven cost?
23: A purse and a mixing bowl together cost $5.40. The purse costs $5 more than the mixing

bowl. How much does the mixing bowl cost?
24: A hat and a mouse together cost $540. The hat costs $500 more than the mouse. How much does the mouse cost?
25: A purse and a box of cigarettes together cost $1.40. The purse costs $1 more than the box of cigarettes. How much does the box of cigarettes cost?
26: A box of mascara and a microwave oven together cost $14. The box of mascara costs $10 more than the microwave oven. How much does the microwave oven cost?
27: A bag and a food processor together cost $54. The bag costs $50 more than the food processor. How much does the food processor cost?
28: A coat and a pot together cost $510. The coat costs $500 more than the pot. How much does the pot cost?
29: A jacket and a set of wine glasses together cost $5.10. The jacket costs $5 more than the set of wine glasses. How much does the set of wine glasses cost?
30: A hat and a coffee table together cost $1.20. The hat costs $1 more than the coffee table. How much does the coffee table cost?
31: A pencil and a keyboard together cost $14. The pencil costs $10 more than the keyboard. How much does the keyboard cost?
32: A bag and a coffee table together cost $53. The bag costs $50 more than the coffee table. How much does the coffee table cost?
33: A mixing bowl and a laundry detergent together cost $52. The mixing bowl costs $50 more than the laundry detergent. How much does the laundry detergent cost?
34: A perfume and a bottle of ouzo together cost $120. The perfume costs $100 more than the bottle of ouzo. How much does the bottle of ouzo cost?
35: A bottle of bath salts and a pack of diapers together cost $510. The bottle of bath salts costs $500 more than the pack of diapers. How much does the pack of diapers cost?
36: A CD and a pan together cost $13. The CD costs $10 more than the pan. How much does the pan cost?
37: A rice cooker and a sculpture together cost $130. The rice cooker costs $100 more than the sculpture. How much does the sculpture cost?
38: A pair of sunglasses and a toy together cost $13. The pair of sunglasses costs $10 more than the toy. How much does the toy cost?
39: A speaker and a bottle of ouzo together cost $5.20. The speaker costs $5 more than the bottle of ouzo. How much does the bottle of ouzo cost?
40: A set of crayons and a wallet together cost $5.20. The set of crayons costs $5 more than the wallet. How much does the wallet cost?
41: A rug and a sculpture together cost $54. The rug costs $50 more than the sculpture. How much does the sculpture cost?
42: A pair of sunglasses and a light bulb together cost $130. The pair of sunglasses costs $100 more than the light bulb. How much does the light bulb cost?
43: A scarf and a ring together cost $1.20. The scarf costs $1 more than the ring. How much does the ring cost?
44: A pair of leggings and a scarf together cost $52. The pair of leggings costs $50 more than the scarf. How much does the scarf cost?
45: A wall clock and a light bulb together cost $1.30. The wall clock costs $1 more than the light bulb. How much does the light bulb cost?
46: A blanket and a flashlight together cost $52. The blanket costs $50 more than the flashlight.

How much does the flashlight cost?
47: A keyboard and a pair of socks together cost $54. The keyboard costs $50 more than the pair of socks. How much does the pair of socks cost?
48: A set of wine glasses and a pizza together cost $53. The set of wine glasses costs $50 more than the pizza. How much does the pizza cost?
49: A pear and a bottle of ouzo together cost $54. The pear costs $50 more than the bottle of ouzo. How much does the bottle of ouzo cost?
50: A pizza and a toy together cost $13. The pizza costs $10 more than the toy. How much does the toy cost?

List of CRT 2 tasks

1: How long does it take 4 people to tailor 4 jackets, if it takes 7 people 7 hours to tailor 7 jackets?

2: How long does it take 4 washing machines to wash 4 loads of laundry, if it takes 8 washing machines 8 hours to wash 8 loads of laundry?

3: How long does it take 50 bees to pollinate 50 flowers, if it takes 60 bees 60 minutes to pollinate 60 flowers?

4: How long does it take 1 carpenter to make 1 chair, if it takes 5 carpenters 5 days to make 5 chairs?

5: How long does it take 10 ovens to bake 10 lasagnas, if it takes 60 ovens 60 minutes to bake 60 lasagnas?

6: How long does it take 1 researcher to publish 1 paper, if it takes 6 researchers 6 years to publish 6 papers?

7: How long does it take 30 cleaners to clean 30 rooms, if it takes 50 cleaners 50 hours to clean 50 rooms?

8: How long does it take 40 students to change 40 light bulbs, if it takes 70 students 70 minutes to change 70 light bulbs?

9: How long does it take 40 builders to build 40 houses, if it takes 50 builders 50 weeks to build 50 houses?

10: How long does it take 5 people to plant 5 trees, if it takes 6 people 6 minutes to plant 6 trees?

11: How long does it take 30 coffee machines to make 30 coffees, if it takes 40 coffee machines 40 minutes to make 40 coffees?

12: How long does it take 5 machines to pack 5 boxes of chocolates, if it takes 8 machines 8 minutes to pack 8 boxes of chocolates?

13: How long does it take 10 children to eat 10 boxes of chocolates, if it takes 50 children 50 minutes to eat 50 boxes of chocolates?

14: How long does it take 2 people to read 2 books, if it takes 4 people 4 weeks to read 4 books?

15: How long does it take 5 teams to renovate 5 houses, if it takes 8 teams 8 weeks to renovate 8 houses?

16: How long does it take 30 people to knit 30 pairs of socks, if it takes 40 people 40 weeks to knit 40 pairs of socks?

17: How long does it take 40 people to pick 40 fields of strawberries, if it takes 70 people 70 hours to pick 70 fields of strawberries?

18: How long does it take 6 programmers to write 6 lines of code, if it takes 7 programmers 7 hours to write 7 lines of code?

19: How long does it take 4 photographers to take 4 photos, if it takes 8 photographers 8 hours to take 8 photos?

20: How long does it take 1 painter to paint 1 painting, if it takes 8 painters 8 hours to paint 8 paintings?

21: How long does it take 50 writers to write 50 books, if it takes 70 writers 70 minutes to write 70 books?

22: How long does it take 2 cooks to cook 2 meals, if it takes 8 cooks 8 minutes to cook 8 meals?

23: How long does it take 50 doctors to examine 50 patients, if it takes 60 doctors 60 minutes to examine 60 patients?

24: How long does it take 2 drivers to change 2 tires, if it takes 7 drivers 7 minutes to change 7 tires?

25: How long does it take 2 farm workers to pick 2 apples, if it takes 8 farm workers 8 seconds to pick 8 apples?
26: How long does it take 4 freezers to freeze 4 liters of water, if it takes 6 freezers 6 hours to freeze 6 liters of water?
27: How long does it take 20 bakers to bake 20 cakes, if it takes 80 bakers 80 hours to bake 80 cakes?
28: How long does it take 30 hair stylists to finish 30 hairstyles, if it takes 50 hair stylists 50 minutes to finish 50 hairstyles?
29: How long does it take 2 mechanics to fix 2 cars, if it takes 3 mechanics 3 hours to fix 3 cars?
30: How long does it take 20 tailors to make 20 dresses, if it takes 50 tailors 50 hours to make 50 dresses?
31: How long does it take 3 painters to paint 3 rooms, if it takes 4 painters 4 hours to paint 4 rooms?
32: How long does it take 40 trees to grow 40 leaves, if it takes 60 trees 60 days to grow 60 leaves?
33: How long does it take 1 runner to clean 1 shoe, if it takes 5 runners 5 minutes to clean 5 shoes?
34: How long does it take 3 translators to translate 3 pages, if it takes 6 translators 6 hours to translate 6 pages?
35: How long does it take 2 machines to make 2 smartphones, if it takes 3 machines 3 hours to make 3 smartphones?
36: How long does it take 50 people to smoke 50 cigarettes, if it takes 70 people 70 minutes to smoke 70 cigarettes?
37: How long does it take 7 opticians to make 7 glasses, if it takes 8 opticians 8 days to make 8 glasses?
38: How long does it take 40 pipes to fill 40 containers, if it takes 60 pipes 60 hours to fill 60 containers?
39: How long does it take 50 people to eat 50 pizzas, if it takes 80 people 80 minutes to eat 80 pizzas?
40: How long does it take 20 kettles to boil 20 liters of water, if it takes 80 kettles 80 hours to boil 80 liters of water?
41: How long does it take 40 air conditioners to cool 40 rooms, if it takes 80 air conditioners 80 minutes to cool 80 rooms?
42: How long does it take 5 students to finish 5 exams, if it takes 7 students 7 minutes to finish 7 exams?
43: How long does it take 40 men to make 40 pies, if it takes 70 men 70 minutes to make 70 pies?
44: How long does it take 1 barista to make 1 coffee, if it takes 8 baristas 8 minutes to make 8 coffees?
45: How long does it take 10 people to cook 10 packs of spaghetti, if it takes 70 people 70 minutes to cook 70 packs of spaghetti?
46: How long does it take 5 people to renovate 5 bathrooms, if it takes 8 people 8 days to renovate 8 bathrooms?
47: How long does it take 1 fish to eat 1 worm, if it takes 3 fish 3 days to eat 3 worms?
48: How long does it take 1 operator to connect 1 phone call, if it takes 3 operators 3 hours to connect 3 phone calls?

49: How long does it take 20 printers to print 20 documents, if it takes 80 printers 80 minutes to print 80 documents?

50: How long does it take 70 husbands to feed 70 babies, if it takes 80 husbands 80 minutes to feed 80 babies?

List of CRT 3 tasks

1: In a city, a virus is spreading, causing the total number of infected individuals to double each day. If it takes 6 days for the entire city's population to be infected, how many days would it require for half of the people to become infected?

2: A pandemic is occurring in a state where the total number of infected individuals doubles daily. If it takes 10 days for the entire state's population to become infected, how many days would it take for half of the state's population to be infected?

3: People are escaping from war. Each day, the total count of refugees doubles. If it takes 22 days for the entire population to evacuate, how long would it take for half of the population to do so?

4: A farmer is plowing a field. Each hour, the total plowed area doubles. If it takes 10 hours for the entire field to be plowed, how long would it take for half of the field to be plowed?

5: The apples are dropping from an apple tree, with the total count of fallen apples doubling each day. If it requires 16 days for all the apples to drop, how many days would it take for half the apples to fall?

6: Fish are migrating and each day, the total distance they cover doubles. If it takes the fish 18 days to reach their destination, how many days would it take for them to cover half the distance?

7: A tree branch is falling, and with each passing second, the total distance it covered doubles. If it takes 6 seconds for the branch to reach the ground, how long would it take for it to cover one-half of the total distance?

8: A fly is traveling from point A to point B. With each passing hour, the total distance it covered doubles. If the fly reaches point B in 12 hours, how long does it take for the fly to cover half of the distance?

9: A new concrete pavement is drying. The overall area of dried concrete doubles each day. If it requires four days for the entire pavement to dry completely, how many days does it take for half the pavement to become dry?

10: There is a freezer filled with food, and the total volume of the frozen food doubles every hour. If it takes 16 hours for all the food to become frozen, how long would it take to freeze half of the food?

11: A pot of water is boiling on the stove, and with each passing hour, the overall volume of the evaporated water doubles. If the entire pot takes 6 hours to evaporate completely, how long does it take for half of the pot to evaporate?

12: There is a section of mold on a bread loaf that doubles in size every hour. If it takes 16 hours for the mold to completely cover the bread, how much time is needed for the mold to cover half of the bread?

13: A forest is engulfed in flames. Each day, the overall area of the scorched forest doubles in size. If it takes 18 days for the entire forest to be consumed by the fire, how many days would it take for half of the forest to be burnt?

14: In a cave, there is a colony of bats with a daily population doubling. Given that it takes 60 days for the entire cave to be filled with bats, how many days would it take for the cave to be half-filled with bats?

15: A section of grass is expanding within a garden, with the total area it occupies doubling daily. If it requires 12 days for the entire garden to be encompassed by the grass, how many days are needed for the grass to cover half of the garden?

16: An investor possesses 1 bitcoin. Each day, their number of bitcoins doubles. If it takes them 30 days to achieve their investment target, how long would it take for them to reach half of that

target?
17: Fish inhabit a creek, and their population doubles each week. If it requires 24 weeks for the entire creek to become completely filled with fish, how long would it take to fill half of the creek with fish?
18: A dust cloud hovers above the city, doubling in size each day. If it takes 12 days for the entire city to be engulfed by the cloud, how many days does it take for the cloud to cover half of the city?
19: There is a sick student in the class. Each day, the number of sick students doubles. If it takes 6 days for the entire class to become sick, how many days does it take for half of the class to become sick?
20: A colony of bacteria is growing on yogurt, and the size of the colony doubles daily. If it takes 4 days for the colony to completely cover the yogurt, how many days does it take for the patch to cover half of the yogurt?
21: A moss patch is expanding on a rock, doubling its size each day. It takes 300 days for the moss to completely cover the rock. How many days are required for the moss to cover half of the rock?
22: Beneath a tree, there is a heap of leaves that doubles in size every week. If it takes four weeks for the pile to attain a height of 4 meters, how much time is required for it to reach a height of 2 meters?
23: Mushrooms are cultivated in a container, and their quantity doubles daily. Given that it takes 6 days for the mushrooms to fill the entire container, determine the time needed to fill half of the container.
24: There is a man who raises rabbits in a barn. Each year, the rabbit population doubles. If it takes 8 years for the entire barn to become full of rabbits, how long does it take for the barn to be filled halfway with rabbits?
25: Within a forest, there is a growing patch of ramson that doubles in size each week. If it takes 10 weeks for the entire forest to be covered with ramson, how long would it take for the ramson to cover half of the forest?
26: It is currently raining, causing the lake to fill up with water. The volume of water in the lake doubles every day. If it takes a total of 20 days for the lake to become completely filled, how many days would it take for the lake to reach halfway to being full?
27: Within a forest, there exists a tree that doubles its height each year. If the tree attains its maximum height in 10 years, determine the time it would take to achieve half of that maximum height.
28: There is a flood occurring in a field. With each passing hour, the size of the flood-stricken area doubles. If it takes 20 hours for the entire field to become submerged, how many hours would it take for half of the field to be inundated?
29: A barrel is being filled with whiskey, and the total volume of whiskey doubles every minute. If it takes 12 minutes to completely fill the barrel, how long would it take to fill half of it with whiskey?
30: Programmers are in the process of developing new software and each month the total quantity of written code doubles. If it requires 10 months to complete the entire code, how much time is needed to write half of the code?
31: A factory is busy filling bags with chocolate cookies. The total number of bags filled doubles with each passing hour. Given that it takes 8 hours to fill all the bags, how much time is required to fill half of the bags?

32: An orange tree is sprouting leaves. The number of leaves doubles every month. Given that it takes six months for the entire tree to be covered with leaves, how many months does it take for the tree to be half covered with leaves?
33: An iceberg is forming and its surface doubles every year. If the iceberg takes 10 years to grow to 10 square miles, how many years are required for it to grow to 5 square miles?
34: A woman is in the process of growing her hair. Each year, the length of her hair doubles. If it takes six years for her hair to achieve a length of two meters, how long would it take for it to reach a length of one meter?
35: Ants are crawling on a cake, and with each passing minute, their population on the cake doubles. If the entire cake is covered with ants in 30 minutes, how long would it take for half of the cake to be covered with ants?
36: In a room, there are rats whose population doubles each month. If it takes 9 months for the rats to completely fill the room, how long would it take for them to occupy half of the room?
37: The wood is burning in a fireplace, and the temperature doubles every minute. If it takes 20 minutes for the fireplace to reach a temperature of 600 degrees, how long does it take for the temperature to reach 300 degrees?
38: In a fish tank, some algae are present. Each day, the quantity of algae multiplies by two. If it requires 30 days for the entire fish tank to become filled with algae, how much time is needed for half of the fish tank to be filled with algae?
39: An elderly woman regularly feeds cats. Each day, the number of cats she feeds doubles. If she feeds 64 cats on the 6th day, on which day does she feed 32 cats?
40: Bamboo is growing in the garden, and its height doubles each day. It takes 5 days to reach a height of 10 meters. How long does it take for the bamboo to attain a height of 5 meters?
41: A patient has been diagnosed with cancer, and the number of cancer cells doubles each week. If it takes four weeks for the cancer cells to reach a critical amount, how long will it take for the cells to reach half of that critical amount?
42: There is a heap of fruit that is decaying. The quantity of spoiled fruit doubles daily. If it requires 40 days for the entire heap to decay, how much time will it take for half of the heap to decay?
43: During winter, the lake is gradually freezing. Each day, the ice covering the lake's surface doubles in size. If it takes 10 days for the entire surface of the lake to freeze, how long does it take for half of the lake's surface to become frozen?
44: During winter, snowfall is occurring, and the depth of the snow cover doubles every hour. If it takes 10 hours for the snow depth to reach 2 meters, how much time is required for it to reach a depth of 1 meter?
45: Five individuals are constructing a home. Each week, the cumulative amount of bricks they place doubles. Given that it takes them 8 weeks to lay all the bricks, determine the duration required for them to lay half of the bricks.
46: Two painters are working on painting a house. With every hour that passes, the total area they've painted doubles. If it takes 16 hours to paint the entire house, how many hours would it take for them to paint half of the house?
47: A grandmother is knitting a scarf for her grandson. Each week, the scarf's length increases twofold. If it takes six weeks to complete the entire scarf, how long would it take to knit half of the scarf?
48: A forest is expanding on an island, with the area occupied by the trees doubling each year. If it takes 140 years for the entire island to be covered with trees, how many years would it take for

the forest to cover half of the island?
49: A gas cylinder is experiencing a leak. With each passing hour, the total quantity of leaked gas doubles. If it requires 4 hours for the entire gas to leak out, how much time is needed for half of the gas to escape?
50: People are walking into a theater and taking their seats. The number of people in the room multiplies by two every minute. If it takes 6 minutes for all of the seats to be taken, how long does it take for half of the seats to be taken?

List of Semantic Illusions

1: Who is the dictator of South Korea?
2: In which year did Germany win World War II?
3: What kind of animal is Mickey's girlfriend Daisy?
4: Which country did Columbus mistakenly believe he had discovered after sailing across the Pacific?
5: In which year did the Americans liberate the Auschwitz concentration camp in Poland in World War II?
6: In which year did the American astronaut Louis Armstrong walk on the Moon?
7: What do bees produce after they collect wax from flowers?
8: In which country did Martin Luther King spark the Protestant Reformation in 1517?
9: What was the name of the mountain on which Noah received the Ten Commandments?
10: Where on their bodies do whales have their gills?
11: How many dwarves befriended Cinderella?
12: What was the name of the goat that was the first mammal to be cloned?
13: According to the Old Testament, how did Jesus die?
14: Can a man marry his widow's sister?
15: What is the profession of Superman's alter ego Bruce Wayne?
16: What sport is played at Roland Garros in Italy?
17: In the old testament, who were the Jews running from when Noah opened the sea?
18: What word did Epicure shout in his bath when he discovered Archimedes' principle?
19: What did the Mirror reply to Snow White when she asked 'Mirror, mirror on the wall, who is the most beautiful of all'?
20: In what famous novel does Alice state 'I'm late I'm late for a very important date'?
21: What country was Barack Obama the prime minister of?
22: In what month does the capital of Brazil host the famous Rio carnival?
23: In a famous Disney Movie, how many dogs did Ursula need for her Dalmatian fur?
24: How many squares are there on the Olympic logo?
25: What does the Roman letter alpha correspond to in the latin alphabet?
26: What famous space company did Jeff Bezos start after he founded Tesla?
27: What famous international sports event invented by the Romans is held every 4 years?
28: How many parts did the Titanic split into before it sank into the Pacific?
29: For what did Cruella trade the voice of the Little Mermaid?
30: What do the penguins in the Arctic eat?
31: What color are the polar bears in Antarctica?
32: Why did the Titanic sink into the Pacific?
33: What is the name of the process in which plants use chlorophyll to produce CO_2?
34: What country is Sydney the capital of?
35: What US state is San Francisco the capital of?
36: How many blue stars are there on the American flag?
37: In which months do ostriches fly to warmer regions?
38: Why did Pablo Picasso cut off his ear?
39: How many points are there in the Star of Moses, or the Jewish star?
40: Which country was Angela Merkel the president of?
41: Why did George W. Bush invade Iran?
42: Which bird symbolizes and accompanies Minerva, the Greek goddess of wisdom?

43: Who is the architect of the famous Eiffel Tower in Marseille?
44: What did Little Red Riding Hood ask her grandmother before she replied 'All the better to see you with, my dear'?
45: Two nuclear bombs named Fat Boy and Little Man were dropped on which two Japanese cities in World War II?
46: When did Mark Zuckerberg found Instagram?
47: Which famous artist designed the famous church, la Sagrada Familia, located in Madrid?
48: Which country did the soccer player Diego Maradona, aka the Silver Boy, represent?
49: In the Marvel Universe, what is the name of Batman’s sidekick?
50: Which Pakistani city is home to the Taj Mahal?

Questions used to test the knowledge necessary to solve semantic illusions.

1: Is South Korea a dictatorship?
2: Did Germany win World War II?
3: What is the name of the girlfriend of Mickey, the Disney character?
4: What ocean did Columbus cross before discovering America?
5: Which country's army liberated Auschwitz during World War II?
6: What was the name of the American astronaut that first walked on the Moon?
7: What do bees collect from flowers?
8: What was the name of the priest that sparked the Protestant Reformation in 1517?
9: What was the name of the person who received the Ten Commandments on Mount Sinai?
10: Do whales have gills?
11: Which fairy-tale character befriended seven dwarfs?
12: What was the species of the first mammal to be cloned?
13: Is Jesus's crucifixion described in the Old or in the New Testament?
14: What does 'widow' mean?
15: What is the name of Superman's alter ego?
16: In which country is the Roland Garros tournament held?
17: What was the name of the prophet who parted the sea to allow Jews to flee from Egypt?
18: Who discovered Archimedes' principle?
19: What was the name of the character who asked 'Mirror, mirror on the wall, who is the most beautiful of all'?
20: Which famous novel character said "I'm late, I'm late for a very important date"?
21: What was Barack Obama's most famous job?
22: What is the capital of Brazil?
23: Who needed 101 Dalmatians to make a Dalmatian fur in a famous Disney movie?
24: What are the geometric shapes on the Olympic logo?
25: What alphabet does the letter alpha come from?
26: Did Jeff Bezos found Tesla?
27: Which nation invented the Olympic games?
28: Into which Ocean did Titanic sink?
29: With whom did Little Mermaid trade voice for legs?
30: Do penguins live in the Arctic?
31: Do polar bears live in Antarctica?
32: Into which Ocean did Titanic sink?
33: What do plants release when they breathe?
34: What is the capital of Australia?
35: What is the capital of California?
36: What is the color of the stars on the American flag?
37: Can ostriches fly?
38: Which famous artist cut off his ear?
39: What is the name of the Jewish star?
40: What was Angela Merkel's most famous job?
41: Did president Bush invade Iran?
42: Is Minerva a Greek or Roman goddess?
43: In which city is the Eiffel Tower located?
44: Who said 'The better to see you with, my dear' to the Little Red Riding Hood?

45: What were the names of the two nuclear bombs that were dropped on two Japanese cities in World War II?
46: Who founded Instagram?
47: Where is the famous church, la Sagrada Familia, located?
48: What was the nickname of Diego Maradona? (hint: it includes the word 'boy')
49: Who owns the rights to the Batman franchise?
50: In which country is Taj Mahal located?

Study description given to human participants

DESCRIPTION: You are invited to participate in a research study exploring how people think, make decisions, and process language. You will be asked to complete several short tasks via a web browser. Participation in this research is voluntary, and you are free to withdraw your consent at any time.

TIME INVOLVEMENT: Your participation will take approximately 1-5 minutes.

PAYMENTS: You will receive $0.40 as payment for your participation.

PRIVACY AND CONFIDENTIALITY: Your individual privacy will be maintained during the research and in all published and written data resulting from the study. Your identity will remain confidential. will remain fully anonymous.

CONTACT INFORMATION: If you have any questions, concerns or complaints about this research, its procedures, risks and benefits, contact Michal Kosinski at Stanford University (michalk@stanford.edu).

Independent Contact: If you are not satisfied with how this study is being conducted, or if you have any concerns, complaints, or general questions about the research or your rights as a participant, please contact the Stanford Institutional Review Board (IRB) to speak to someone independent of the research team at 650-723-2480 or toll free at 1-866-680-2906, or email at irbnonmed@stanford.edu. You can also write to the Stanford IRB, Stanford University, 1705 El Camino Real, Palo Alto, CA 94306.

Please save or print a copy of this page for your records.

If you agree to participate in this research, please continue.

Please deactivate Ghostery/AdBlocker to load the necessary web pages.

Stanford IRB approval date: 2023-04-27

Expiration date: (Does not expire)
