# Supplementary material for: Human-like intuitive behavior and reasoning biases emerged in large language models but disappeared in ChatGPT
Source: Nat Comput Sci. 2023 Oct 5;3(10):833–8. doi: 10.1038/s43588-023-00527-x (PMC10766525; doi:10.1038/s43588-023-00527-x)
Supplement: Supplementary file 2 — Reporting Summary [file 43588_2023_527_MOESM2_ESM.pdf]

nature portfolio

Corresponding author(s): Michal Kosinski

Last updated by author(s): Sep 1, 2023

Reporting Summary

Nature Portfolio wishes to improve the reproducibility of the work that we publish. This form provides structure for consistency and transparency in reporting. For further information on Nature Portfolio policies, see our Editorial Policies and the Editorial Policy Checklist.

Statistics

For all statistical analyses, confirm that the following items are present in the figure legend, table legend, main text, or Methods section.

n/a	Confirmed	
☐	☒	The exact sample size (n) for each experimental group/condition, given as a discrete number and unit of measurement
☐	☒	A statement on whether measurements were taken from distinct samples or whether the same sample was measured repeatedly
☐	☒	The statistical test(s) used AND whether they are one- or two-sided<br>*Only common tests should be described solely by name; describe more complex techniques in the Methods section.*
☐	☒	A description of all covariates tested
☐	☒	A description of any assumptions or corrections, such as tests of normality and adjustment for multiple comparisons
☐	☒	A full description of the statistical parameters including central tendency (e.g. means) or other basic estimates (e.g. regression coefficient) AND variation (e.g. standard deviation) or associated estimates of uncertainty (e.g. confidence intervals)
☐	☒	For null hypothesis testing, the test statistic (e.g. F, t, r) with confidence intervals, effect sizes, degrees of freedom and P value noted<br>*Give P values as exact values whenever suitable.*
☒	☐	For Bayesian analysis, information on the choice of priors and Markov chain Monte Carlo settings
☒	☐	For hierarchical and complex designs, identification of the appropriate level for tests and full reporting of outcomes
☐	☒	Estimates of effect sizes (e.g. Cohen's d, Pearson's r), indicating how they were calculated

Our web collection on statistics for biologists contains articles on many of the points above.

Software and code

Policy information about availability of computer code

Data collection	Data was collected using OpenAI API, HuggingFace API. Human participants were recruited on Prolific.co
Data analysis	We used GPT-1, GPT-2XL, GPT-3-[ada-001/babbage-001/curie-001/davinci-00[1/2/3]], ChatGPT-[3.5-turbo/4] to generate responses. Data was collected using openai (v. 0.27.1) and requests (v 2.25.1) Python libraries. Data was analysed in Python (3.9.13) and R (4.3.1).

For manuscripts utilizing custom algorithms or software that are central to the research but not yet described in published literature, software must be made available to editors and reviewers. We strongly encourage code deposition in a community repository (e.g. GitHub). See the Nature Portfolio guidelines for submitting code & software for further information.

Data

Policy information about availability of data

All manuscripts must include a data availability statement. This statement should provide the following information, where applicable:

- Accession codes, unique identifiers, or web links for publicly available datasets
- A description of any restrictions on data availability
- For clinical datasets or third party data, please ensure that the statement adheres to our policy

All datasets are publicly available at https://osf.io/w5vhp/.

Human research participants

Policy information about studies involving human research participants and Sex and Gender in Research.

Reporting on sex and gender	We did not collect information about participants' sex or gender.
Population characteristics	50% of participants were female according to Prolific.co
Recruitment	Participants were recruited on Prolific.co
Ethics oversight	The study was reviewed and approved by Stanford University IRB.

Note that full information on the approval of the study protocol must also be provided in the manuscript.

Field-specific reporting

Please select the one below that is the best fit for your research. If you are not sure, read the appropriate sections before making your selection.

☐ Life sciences ☒ Behavioural & social sciences ☐ Ecological, evolutionary & environmental sciences

For a reference copy of the document with all sections, see nature.com/documents/nr-reporting-summary-flat.pdf

Behavioural & social sciences study design

All studies must disclose on these points even when the disclosure is negative.

Study description	A set of 200 reasoning tasks was administered to a range of LLMs and 500 human participants to study the accuracy of their responses.
Research sample	We recruited a sample of 500 anonymous workers on Prolific.co. The manuscript describes the power analysis.
Sampling strategy	Random subset of the US participants (50%/50% male and female) were recruited by the platform.
Data collection	Data was collected on Prolific.co platform. Participants were paid $.40.
Timing	The study was conducted on June 10th, 2023. Participants had max 19 minutes to answer 4 questions (most finished in less than 2 minutes).
Data exclusions	45 participants admitted to using language models to generate their responses and were excluded from the analysis
Non-participation	We only received data from the participants who completed the task and submitted their responses
Randomization	Participants were presented with a random set of 4 tasks.

Reporting for specific materials, systems and methods

We require information from authors about some types of materials, experimental systems and methods used in many studies. Here, indicate whether each material, system or method listed is relevant to your study. If you are not sure if a list item applies to your research, read the appropriate section before selecting a response.

Materials & experimental systems

n/a	Involved in the study	
☒	☐	Antibodies
☒	☐	Eukaryotic cell lines
☒	☐	Palaeontology and archaeology
☒	☐	Animals and other organisms
☒	☐	Clinical data
☒	☐	Dual use research of concern

Methods

n/a	Involved in the study	
☒	☐	ChIP-seq
☒	☐	Flow cytometry
☒	☐	MRI-based neuroimaging
